# Experimental Study on the Compression Characteristics of Sand–Silt–Clay Mixtures

**DOI:** 10.3390/ma18050996

**Published:** 2025-02-24

**Authors:** Tao Li, Jixiao Li, Bingyang Li, Guangtao Yu

**Affiliations:** 1 Key Laboratory of Safety Control of Bridge Engineering, Ministry of Education, Changsha University of Science & Technology, Changsha 410114, China; leejx@stu.csust.edu.cn; 2 School of Civil and Environmental Engineering, Changsha University of Science & Technology, Changsha 410114, China; 3 National Key Laboratory of Green and Long-Life Road Engineering in Extreme Environment, Changsha 410114, China; 4 Elite Engineering School, Changsha University of Science & Technology, Changsha 410114, China; lllbingyang@163.com; 5 School of Traffic & Transportation Engineering, Changsha University of Science & Technology, Changsha 410114, China; yuguangtao163@163.com

**Keywords:** sand–silt–clay mixtures, fines content, clay–silt ratio, compressibility, compression model

## Abstract

In order to investigate the influence of the coexistence of clay and silt on the compression characteristics of sand, one-dimensional compression consolidation tests were carried out on reconstituted saturated sand–silt–clay mixtures with a constant initial void ratio, and the effects of fines content (*FC*) and clay–silt ratio (*CS*) on the compression characteristics of mixed soils were studied. The mechanism of the experimental results was additionally explained from a microscopic perspective. The test results show that: the compressibility of mixed soil increased with the increase in *FC*; the compressibility change rule of mixed soils with different *CS* is consistent under the same *FC*; the influence of *CS* on the *e*–lg*p* (the void ratio (*e*) versus logarithm of the pressure (*p*)) curve of mixed soil is inconsistent when *FC* is different: when *FC* = 3%, the compressibility of mixed soil decreased with the increase in *CS*; when *FC* = 7% and 10%, the compressibility of mixed soil gradually increased with the increase in *CS*; when *FC* = 5%, the compressibility of mixed soil did not show an obvious changing law with the increase in *CS*, and the compressibility of the specimen with *FC* = 5%–*CS* = 1 (*FC* = 5%, *CS* = 1) was the largest; when *CS* was same, the difference between *e*–lg*p* curves of mixed soil with different *FC* increased with the increase in *CS*. The compression model of sand–silt–clay mixtures was established, which can consider the effects of *FC* and *CS*. The reliability and applicability of the proposed model were verified by combining the experimental results of this paper and the test data of sand–clay mixture and sand–silt mixture in other literature.

## 1. Introduction

With the rapid development of engineering construction, increasing demands have been placed on the deformation control of foundation soils for buildings and structures. The deformation behavior of foundation soils is closely related to their compression characteristics. Therefore, the exploration of soil compressibility has become a topic of great interest in the field of geotechnical engineering. At the same time, global renewable energy is continuously advancing, with wind power generation being one of the fastest-growing energy utilization methods. However, compared to onshore wind turbine projects, offshore wind turbine engineering typically faces complex marine environments and specific soil conditions. According to relevant studies, the soil composition in coastal areas is highly complex and exhibits significant regional characteristics [1]. For example, in the coastal region of Hainan Island, China, sandy soils cover a large area and extensive range. Due to seasonal flooding of rivers and the deposition of alluvial materials, these sandy soils are often mixed with silt and clay particles [2]. Therefore, studying the compression characteristics of sand–silt–clay mixtures in coastal areas is of significant importance for engineering construction in these regions. However, existing research on soil compressibility has mainly focused on cohesive soils [3,4] and sandy soils [5,6]. Therefore, there is a need to strengthen the research on the compressibility of sand–silt–clay mixtures. On this issue, some researchers have explored the effect of fines content (*FC*) on the compressibility of sand–silt mixtures [7,8]. It has also been observed that when *FC* is low, coarse particles dominate the properties of the mixed soil. As *FC* increases, the influence of fines particles gradually becomes more pronounced, eventually affecting the one-dimensional compressibility of the mixed soil [9]. Additionally, extensive studies have been conducted on the compressibility characteristics of sand–clay mixtures [10,11,12], revealing that compression of mixed soils can lead to the formation of a sand skeleton within the soil. Once this sand skeleton is established, the compressibility of the soil is primarily controlled by sand particles [10]. Furthermore, the frictional resistance generated at the contact points between sand skeleton particles may reduce the settlement of the mixed soil [12].

In summary, numerous scholars have investigated the compressibility of mixed soils and achieved significant progress. However, current research is primarily focused on sand–clay and sand–silt mixtures, while studies addressing the impact of the coexistence of clay and silt particles on the compressibility of sand—particularly in widely distributed sand–silt–clay mixtures encountered in engineering practice—remain limited. To address this gap, this study aims to conduct experimental research on the influence of coexistence of clay and silt on the compressibility characteristics of sand. Based on experimental observations, the results are further explained through a micro-mechanical perspective. Finally, a compressibility model for sand–silt–clay mixtures is developed, incorporating the effects of fines content (*FC*) and clay–silt ratio (*CS*, defined as the ratio of clay content to silt content). The proposed model is validated using experimental results from this study and data from the related literature [11,13].

## 2. Overview of the Experiments

**Figure 1:**
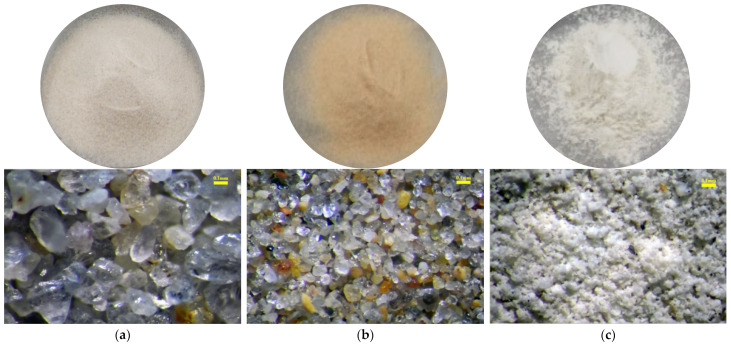
Macroscopic and mesoscopic image of sand, silt and clay: (**a**) sand particles, (**b**) silt particles, and (**c**) clay particles.

The sand particles used in the experiments are Fujian standard sand with particle sizes ranging from 0.1 to 0.25 mm. The silt particles are natural silt with particle sizes between 0.02 and 0.075 mm, and the clay particles are commercially available calcium-based bentonite (WANCHENG BENTONITE, Jinzhou, China). Here, the sand, silt and clay particles are not come from real seabed materials. They are only used to simulate the particle composition of seabed materials. The basic physical parameters of three test materials are summarized in Table 1, and their macroscopic and microscopic morphologies are shown in Figure 1. As observed in Figure 1, the sand and silt particles used in the experiments are nearly spherical with minimal angularity, while the clay particles exhibit a plate-like morphology, often forming aggregated clusters.

## 3. Experimental Plan

**Figure 2:**
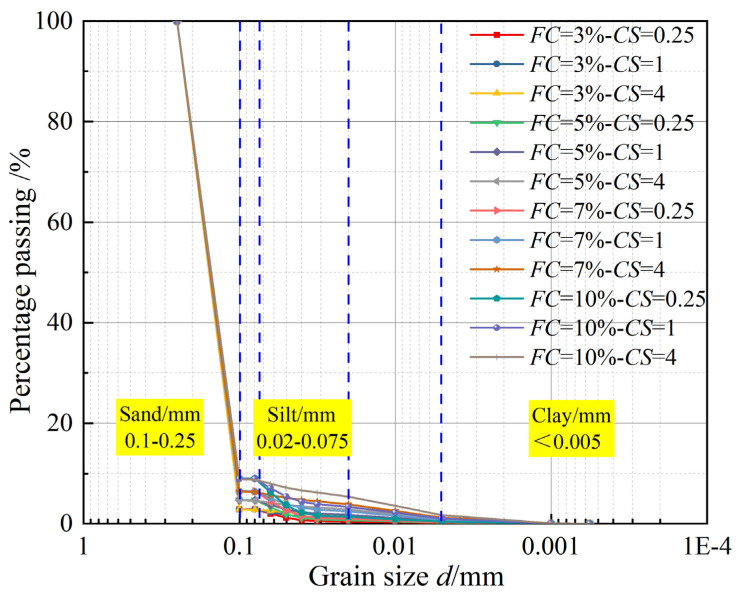
Grain size distribution curves of sand–silt–clay mixtures.

Under a constant initial void ratio (*e*_0_), one-dimensional compression consolidation tests were conducted on remolded sand–silt–clay mixture specimens with varying fines content (*FC* = 3%, 5%, 7%, and 10%) and clay–silt ratio (*CS* = 0.25, 1, and 4). The grain size distribution curves for each mixed soil are presented in Figure 2. The detailed experimental plan is shown in Table 2, and fines content (*FC*), clay content (*CC*), silt content (*SC*) and clay–silt ratio (*CS*) are defined as follows:

(1) $$FC=m_{f}/m_{s}$$

(2) $$CC=m_{c}/m_{s}$$

(3) $$SC=m_{\mathrm{ss}}/m_{s}$$

(4) CS=CC/SC

In the equation, *m*_f_ is the mass of fines (clay and silt), *m*_s_ is the mass of sand, *m*_c_ is the mass of clay, *m*_ss_ is the mass of silt.

**Figure 3:**
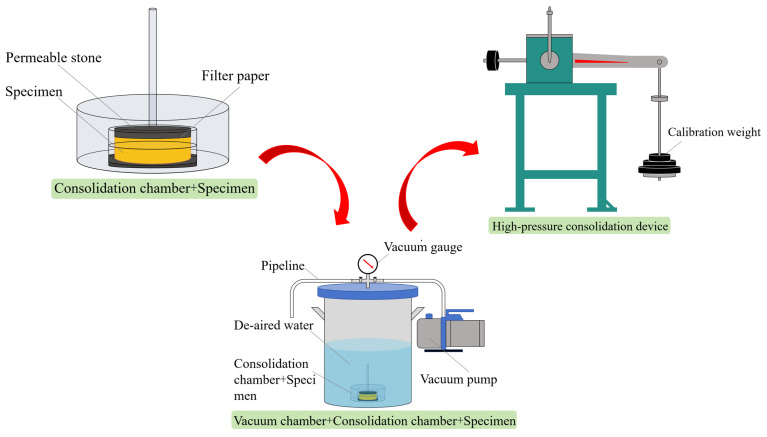
Saturation and loading process of sand–silt–clay mixture specimens.

The specimen dimensions were 61.8 mm × 20 mm (diameter × height). Given the relatively small volume of the specimens used in this experiment, the required quantities of sand, silt, and clay for each specimen were weighed in a single batch based on the specified fines content and clay–silt ratio. These particles were first thoroughly dried and mixed to ensure uniformity, after which 6% de-aired water was added to the dry mixture. The resulting blend was mixed again to achieve homogeneity, and the specimen was subsequently prepared using a one-step compression molding technique. Due to the small height-to-diameter ratio of the specimens and the low cohesion of the mixed soil used in this study, the specimens could not be demolded after preparation, as is typically performed with triaxial test specimens. Additionally, unlike triaxial test specimens, one-dimensional compression consolidation test specimens cannot be saturated directly in the testing apparatus due to equipment limitations. To address these constraints, the prepared specimens were first placed into a consolidation container. The “consolidation container and specimen” assembly was then placed in a vacuum chamber for saturation under vacuum conditions. After saturation, the saturated specimens, along with their consolidation containers, were transferred to the GDG high-pressure consolidation apparatus to complete the subsequent consolidation and compression testing. The detailed process is illustrated in Figure 3. Each specimen was subjected to incremental loading at pressures of 12.5 kPa, 25 kPa, 50 kPa, 100 kPa, 200 kPa, 400 kPa, 800 kPa, and 1600 kPa. The criterion for compression stability was defined as a vertical displacement rate of ≤0.01 mm/h [14]. All testing procedures were conducted in strict accordance with the Standard for Geotechnical Testing Method [14].

## 4. Test Results and Discussion

**Figure 4:**
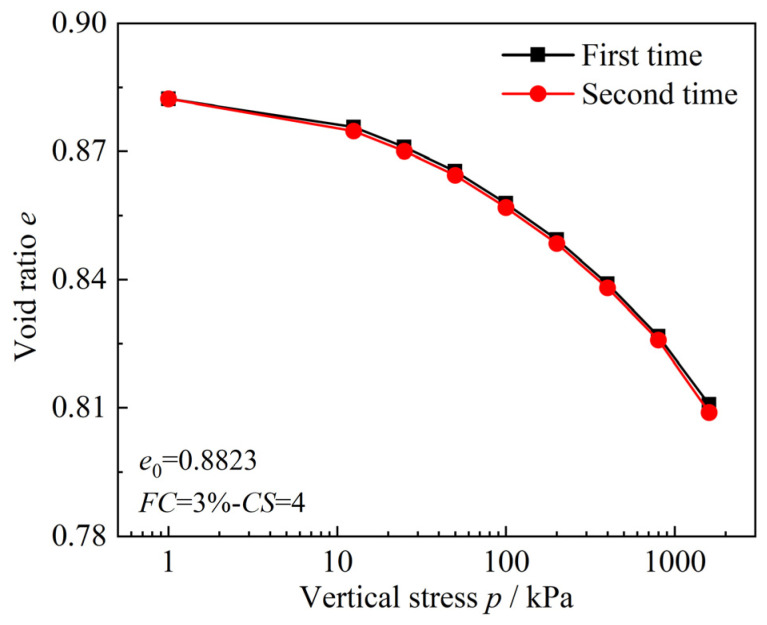
Comparison of two one-dimensional compression consolidation test results of sand–silt–clay mixtures (*FC* = 3%–*CS* = 4).

To verify the repeatability of the specimen preparation method and the experimental procedure in the one-dimensional compression consolidation test, this study selected a specimen with *FC* = 3%–*CS* = 4 to conduct two parallel one-dimensional compression consolidation tests. The experimental results are shown in Figure 4.

From Figure 4, it can be observed that two test results align well, with the compression curves of specimens under various load levels exhibiting a high degree of overlap. This fully demonstrates the repeatability of the specimen preparation method and the experimental procedure used in this study.

**Figure 5:**
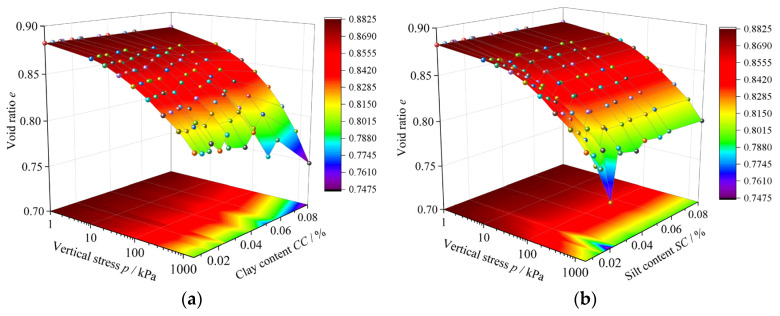
One-dimensional compression consolidation test results of sand–silt–clay mixtures: (**a**) *e–p–CC* relationship curve; (**b**) *e–p–SC* relationship curve.

Figure 5 shows the one-dimensional compression consolidation test results for different sand–silt–clay mixtures. In Figure 5a, the results are plotted with void ratio (*e*), vertical stress (*p*) and clay content (*CC*) as the axes. While in Figure 5b, the results are plotted with void ratio (*e*), vertical stress (*p*) and silt content (*SC*) as the axes. The *CC* and *SC* in the figures represent the absolute clay content and silt content in specimens, respectively. Here, all specimens had the same initial void ratio. It can be observed that under the influence of *p*, the compressive consolidation characteristics of specimens are different. Specifically, when *CC* is higher or *SC* is lower, the specimen undergoes more significant compression. The following discussion will focus on the test results for specimens with same clay–silt ratio (*CS*) and same fines content (*FC*).

### 4.1. The Impact of Fines Content on the Compressibility Characteristics of Sand–Silt–Clay Mixtures

**Figure 6:**
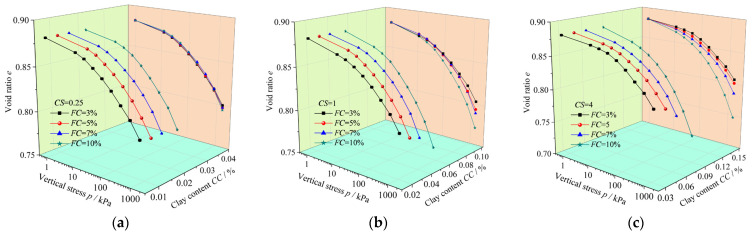
One-dimensional compression consolidation test results of mixed soils with same *CS* and different *FC*: (**a**) *CS* = 0.25; (**b**) *CS* = 1; (**c**) *CS* = 4.

To investigate the effect of fines content (*FC*) on the compressibility characteristics of mixed soils under constant initial void ratio, the test results of specimens with same clay–silt ratio (*CS*) but different fines content (*FC*) were projected onto the same clay content plane (the orange plane in Figure 6). The experimental results on this plane correspond to the conventional two-dimensional *e*–lg*p* curves, with detailed results shown in Figure 6.

**Figure 7:**
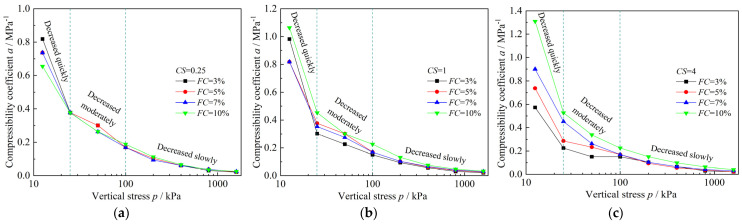
Compressibility coefficient results of mixed soils with same *CS* and different *FC*: (**a**) *CS* = 0.25; (**b**) *CS* = 1; (**c**) *CS* = 4.

As seen in Figure 6, for specimens with same clay–silt ratio (*CS*), the compressibility of specimens generally increases with increasing fines content (*FC*). The results of compressibility coefficient (*a*) (Figure 7) further support this observation, In Figure 7, for each *CS*, the compressibility coefficient increases overall with increasing *FC*. When *CS* = 0.25, the increase is relatively weak, whereas for *CS* = 1 and 4, the compressibility coefficient shows a very pronounced increasing trend with higher *FC*. Additionally, as the *CS* increases, the differences between *e*–lg*p* (the void ratio (*e*) versus logarithm of the pressure (*p*)) curves for specimens with different *FC* become more pronounced (Figure 6), and the corresponding differences in compressibility coefficient also grow larger (Figure 7). Furthermore, from Figure 7 it can be observed that the development of the compressibility coefficient for each specimen can be divided into three stages: a quick decrease stage (12.5 kPa to 25 kPa), a moderate decrease stage (25 kPa to 100 kPa), and a slow decrease stage (100 kPa to 1600 kPa). This indicates that the compressibility coefficient is not constant throughout the consolidation process.

### 4.2. The Impact of Clay–Silt Ratio on the Compressibility Characteristics of Sand–Silt–Clay Mixtures

**Figure 8:**
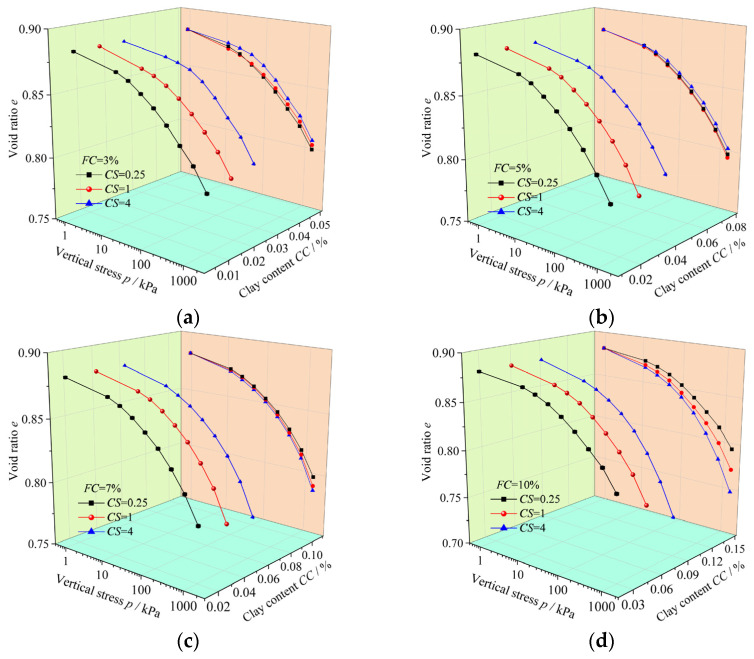
One-dimensional compression consolidation test results of mixed soils with same *FC* and different *CS*: (**a**) *FC* = 3%; (**b**) *FC* = 5%; (**c**) *FC* = 7%; (**d**) *FC* = 10%.

To investigate the effect of clay–silt ratio (*CS*) on the compressibility characteristics of mixed soils under constant initial void ratio (*e*_0_), using the same method as described earlier, the test results of specimens with same fines content but different clay–silt ratio were projected onto the same clay content plane (the orange plane in Figure 8). The experimental results on this plane correspond to the conventional two-dimensional *e*–lg*p* compression curves, with detailed results shown in Figure 8.

**Figure 9:**
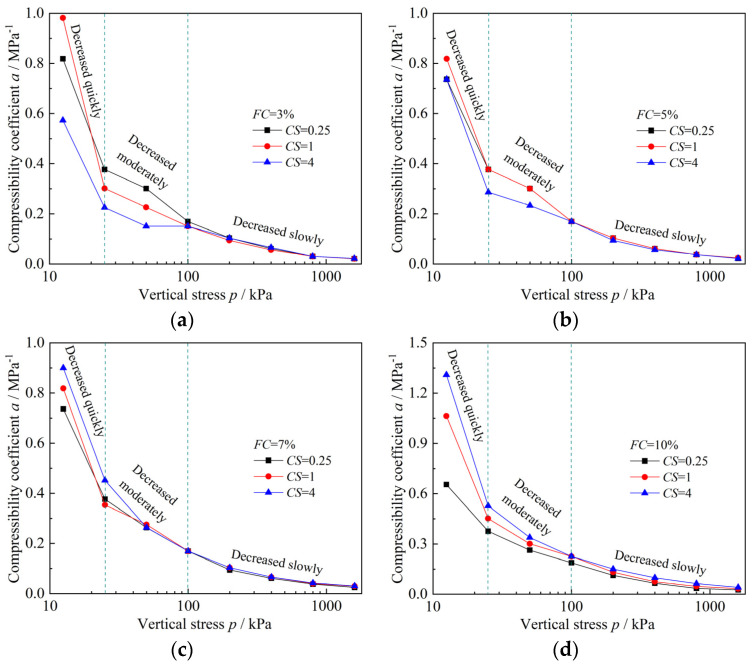
Compressibility coefficient result of mixed soils with same *FC* and different *CS*: (**a**) *FC* = 3%; (**b**) *FC* = 5%; (**c**) *FC* = 7%; (**d**) *FC* = 10%.

As shown in Figure 8, the effect of clay–silt ratio (*CS*) on *e*–lg*p* (the void ratio (*e*) versus logarithm of the pressure (*p*)) curves of mixed soil varies with fines content (*FC*). Specifically: when *FC* = 3% (Figure 8a), as *CS* increases, the compressibility of the specimen gradually decreases. The *e*–lg*p* curve for the specimen with *FC* = 3%–*CS* = 0.25 (*FC* = 3%, *CS* = 0.25) is at the bottom, corresponding to the highest compressibility coefficient, while the *e*–lg*p* curve for the specimen with *FC* = 3%–*CS* = 4 is at the top, corresponding to the lowest compressibility coefficient. When *FC* = 7% (Figure 8c) and *FC* = 10% (Figure 8d), as *CS* increases, the compressibility of the specimen continuously increases. When *FC* = 5% (Figure 8b), there is no clear trend in the compressibility of the specimen as *CS* increases. For this fines content, the specimen with *FC* = 5%–*CS* = 1 exhibits the strongest compressibility. From the compressibility coefficient results shown in Figure 9, similar trends are observed: when *FC* = 3% (Figure 9a), the compressibility coefficient decreases as *CS* increases. When *FC* = 7% and *FC* = 10% (Figure 9c,d), the compressibility coefficient increases as *CS* increases. When *FC* = 5% (Figure 9b), the compressibility coefficient does not exhibit a clear trend with increasing *CS*, and the specimen with *FC* = 5%–*CS* = 1 has the highest compressibility coefficient. For the specimens with *FC* = 7% and *FC* = 10%, as *FC* increases, the differences in the *e*–lg*p* curves between specimens with different *CS* become more pronounced (Figure 8c,d), and the corresponding differences in the compressibility coefficient values also increase (Figure 9c,d).

### 4.3. Mechanism Analysis of the Compressibility Characteristics of Sand–Silt–Clay Mixtures

**Figure 10:**
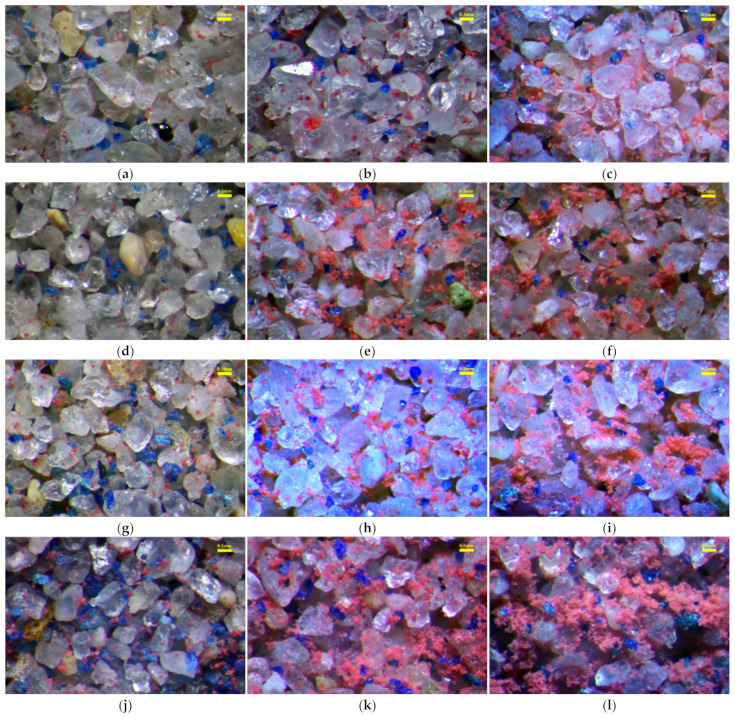
Mesoscopic feature images of sand–silt–clay mixtures (40×): (**a**) *FC* = 3%–*CS* = 0.25; (**b**) *FC* = 3%–*CS* = 1; (**c**) *FC* = 3%–*CS* = 4; (**d**) *FC* = 5%–*CS* = 0.25; (**e**) *FC* = 5%–*CS* = 1; (**f**) *FC* = 5%–*CS* = 4; (**g**) *FC* = 7%–*CS* = 0.25; (**h**) *FC* = 7%–*CS* = 1; (**i**) *FC* = 7%–*CS* = 4; (**j**) *FC* = 10%–*CS* = 0.25; (**k**) *FC* = 10%–*CS* = 1; (**l**) *FC* = 10%–*CS* = 4.

**Figure 11:**
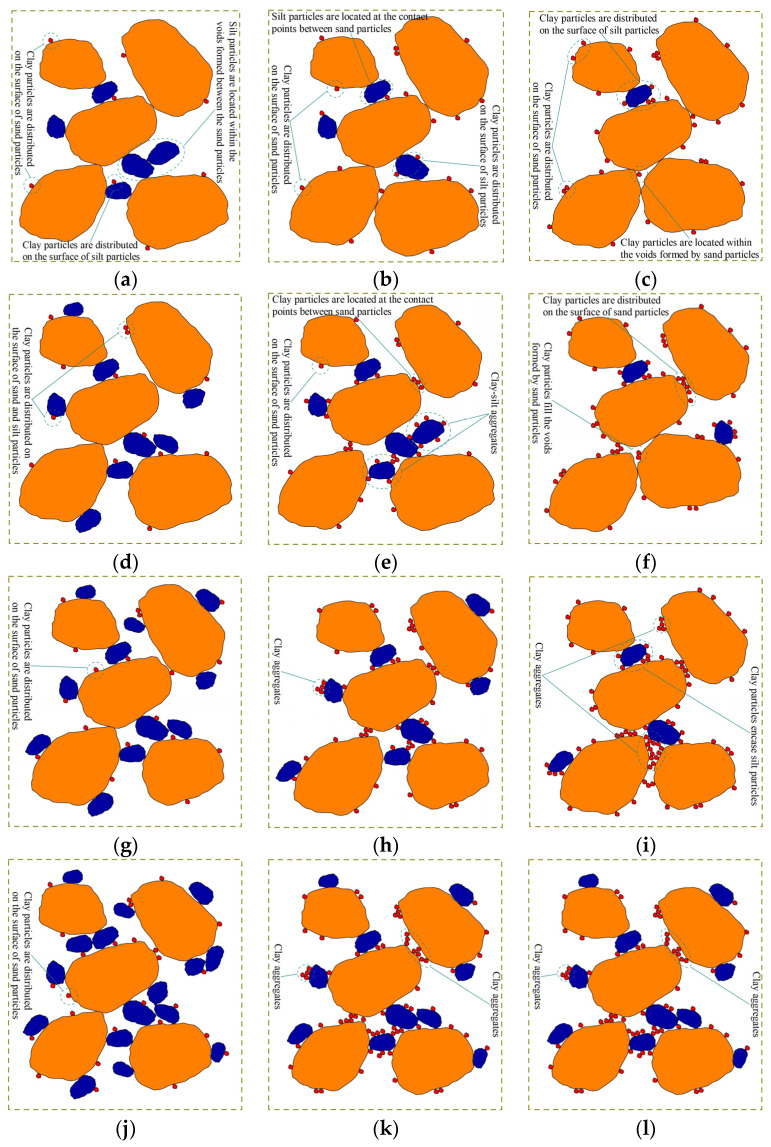
Contact state model of sand–silt–clay mixtures: (**a**) *FC* = 3%–*CS* = 0.25; (**b**) *FC* = 3%–*CS* = 1; (**c**) *FC* = 3%–*CS* = 4; (**d**) *FC* = 5%–*CS* = 0.25; (**e**) *FC* = 5%–*CS* = 1; (**f**) *FC* = 5%–*CS* = 4; (**g**) *FC* = 7%–*CS* = 0.25; (**h**) *FC* = 7%–*CS* = 1; (**i**) *FC* = 7%–*CS* = 4; (**j**) *FC* = 10%–*CS* = 0.25; (**k**) *FC* = 10%–*CS* = 1; (**l**) *FC* = 10%–*CS* = 4.

Based on the test results, the patterns of sand–silt–clay mixtures, and in conjunction with the microstructural images of sand–silt–clay mixtures (Figure 10) [15], this study further establishes a particle contact state model for sand–silt–clay mixtures (Figure 11). In Figure 10, optical microscopy and particle dyeing techniques has been used in the early research of author to study the internal contact status of sand–silt–clay mixtures, in which 40× magnification was used. And in Figure 10, transparent particles represent sand particles, blue particles represent dyed silt particles, and red particles represent dyed clay particles. In Figure 11, yellow particles represent sand particles, blue particles represent silt particles, and red particles represent clay particles.

From Figure 10 and Figure 11, it can be observed that when the clay content is low, clay particles are mostly distributed on the surfaces of sand or silt particles, as well as within the pores formed by sand particles. A small portion of clay particles is located at the contact points between adjacent sand particles. Although previous studies have indicated that fines particles located at the contact points of coarse particles have a greater effect on the soil properties compared to those within the pores [16], when the clay content is too low, the clay particles cannot effectively participate in the formation of the soil framework [17], or the role played by clay particles at the contact points of sand particles is extremely limited. As a result, the effect of clay particles on the compressibility of the soil can be neglected, and the compressibility is primarily controlled by the sand particles. In addition, silt particles have a relatively high internal friction angle [18], and are not easily compressible. Therefore, during specimen preparation, the presence of silt particles makes it difficult to form a compact and stable structure within the specimen.

As a result, sand–silt mixtures exhibit greater compressibility in one-dimensional compression consolidation tests. In this case, the compressibility of the mixed soil is jointly determined by both sand and silt particles. For fines content (*FC*) of 3%, the content of clay and silt particles is relatively low. A portion of the silt particles fills the pores formed by the sand particles, while another portion is located at the contact points between the sand particles, where it works together with the sand particles to contribute to the soil framework. These silt particles have a significant impact on the compressibility of the soil. Meanwhile, the clay particles are mostly distributed on the surfaces of sand or silt particles, and their effect on the compressibility of the soil is relatively small (Figure 11a–c). Therefore, a comprehensive analysis shows that, in this case, the effect of silt particles on soil compressibility is more significant. This results in a decrease in compressibility as the clay–silt ratio (*CS*) increases (with an increase in clay content and a decrease in silt content) for the specimen with *FC* = 3%. When *FC* = 7% and 10%, as *FC* increases, both the clay and silt contents increase. Compared to the filling and framework effects of silt particles in the sand particle pores, the presence of clay particles in the soil becomes more diverse: ① some clay particles form clay aggregates (Figure 10i,k,l and Figure 11i,k,l); ② some clay particles encase sand and silt particles (Figure 10i,l and Figure 11i,l); ③ some contact points between sand-sand or sand–silt particles are isolated, and in some cases, sand or silt particles are even suspended (Figure 10l and Figure 11l). The friction between the sand and silt particles that are wrapped, isolated, or suspended is reduced, weakening their contribution to soil compressibility. Meanwhile, the clay aggregates formed by some clay particles have larger pores [19], which are more easily compressed, further increasing the compressibility of the soil. Therefore, a comprehensive analysis reveals that, when *FC* = 7% and 10%, compared to silt, clay contributes more to soil compressibility, and its contribution is more varied. As a result, the final experimental results show that as *CS* increases, the compressibility of the mixed soil also increases.

When *FC* = 5%, although the fines content in the specimen increases, it is still relatively low overall, with the fines particles mainly filling the pores of the sand particles. However, the effect varies with the different clay–silt ratio (*CS*). In the specimen with *FC* = 5%–*CS* = 1 (*FC* = 5%, *CS* = 1), where the clay and silt contents are “equivalent”, these two fines particles fill the sand particle pores and bond with each other to form clay-silt aggregates (Figure 10e and Figure 11e). This aggregate combines the individual effects of clay and silt particles and contributes significantly to the soil’s compressibility. Therefore, the specimen with *FC* = 5%–*CS* = 1 exhibits the highest compressibility. In specimens with *FC* = 5%–*CS* = 4 and *FC* = 5%–*CS* = 0.25, the clay and silt particles are in relative excess, and these fines particles, in “equivalent amounts” fill the sand particle pores and bond together. In the specimen with *FC* = 5%–*CS* = 0.25, some of the “excess” silt particles play a role in the soil framework, while the silt particles also enhance the friction between sand particles, which reduces the compressibility of the specimen.

In the specimen with *FC* = 5%–*CS* = 4, the “excess” clay particles are scattered on the surfaces and contact points of the sand particles. These clay particles, to some extent, strengthen the bonding between sand particles, thereby reducing the specimen’s compressibility. Based on the experimental results, it is reasonably inferred that the bonding effect provided by the “excess” clay particles is greater than the frictional effect provided by the “excess” silt particles, which leads to the lowest compressibility in the *FC* = 5%–*CS* = 4 specimen. This analysis suggests that the fines content and the clay–silt ratio in the specimen with *FC* = 5%–*CS* = 1 represent a “threshold” point for the compressibility of the mixed soil. Similar “threshold” points for mixed soil properties have been described in related studies [9,17,20,21,22].

## 5. Establishment and Validation of the Compression Model for Sand–Silt–Clay Mixtures

### 5.1. Establishment of the Compression Model for Sand–Silt–Clay Mixtures

Based on the test results of sand–silt–clay mixtures and their compressibility characteristics above, this study attempts to establish a mathematical model that considers the influence of fines content (*FC*) and clay–silt ratio (*CS*) on the compressibility characteristics of sand–silt–clay mixtures. Ref. [23] pointed out that in isotropic compression tests, the relationship between the volumetric strain of cohesionless soils and the consolidation pressure them is as follows:

(5) $$\varepsilon_{v}=t{\left(p/p_{a}\right)}^{\lambda }$$

In the equation, *ε*_v_ represents the volumetric strain; *p* is the consolidation stress; *p*_a_ is the atmospheric pressure; *t* and *λ* are fitting parameters.

However, through analysis, it can be observed that Equation (5) can only be used to calculate the volumetric deformation or changes in the void ratio during the consolidation of the soil at a given initial density [6]. It does not specifically reflect the influence of *FC* and *CS* on the compression curve. Therefore, in this study, based on the Equation (5), an attempt is made to establish a compression model for mixed soils that incorporates the effects of *FC* and *CS*. As is well known, under unidirectional compression conditions, the volumetric strain (*ε*_v_) of the specimen is actually equal to its vertical strain (*ε*), i.e., *ε*_v_ = *ε*, and the relationship between vertical strain and void ratio can be expressed by the following Equation (6):

(6) $$e=e_{0}-(1+e_{0})\varepsilon$$

In the equation, *e* represents the void ratio, and *e*_0_ is the initial void ratio.

Substituting Equation (5) into Equation (6) further gives:

(7) $$e=e_{0}-k{\left(p/p_{a}\right)}^{\lambda }$$

In the equation, *k* = (1 + *e*_0_) *t*.

Next, Equation (7) is used to fit and analyze the one-dimensional compression consolidation test results of sand–silt–clay mixtures in this study, ultimately obtaining the fitting parameters for mixed soils with different *FC* and *CS*. The results are shown in Table 3.

**Figure 12:**
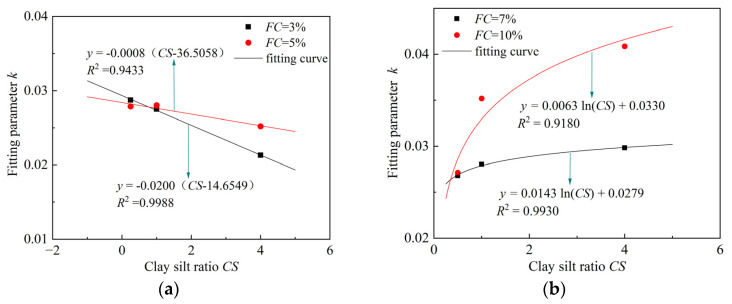
Relationship between *k* and *CS* under different *FC*: (**a**) *FC* = 3%, 5%; (**b**) *FC* = 7%, 10%.

Next, the fitting parameter *k* at different fines contents (*FC*) is plotted against the clay–silt ratio (*CS*) on the same graph, as shown in Figure 12. It can be observed that when *FC* = 3% and 5%, there is a good linear relationship between *CS* and *k* (Figure 12a). However, when *FC* = 7% and 10%, the relationship between *CS* and *k* follows a logarithmic pattern (Figure 12b). The different functional relationships between *CS* and *k* at varying *FC* further confirm the “threshold point” mentioned in the related literature earlier. Based on this observation, function relationships between *CS* and *k* are proposed for *FC* = 3%, 5%, and *FC* = 7%, 10%, as given by Equations (8) and (9), respectively:

(8) $$k=\alpha (CS-CS_{1})$$

In the equations, *α* is the slope of the line, and *CS*_1_ is the reference clay–silt ratio, which corresponds to the intercept of the line with the horizontal axis.

(9) k=βlnCS+δ

In the equation, *β* and *δ* are fitting parameters.

**Figure 13:**
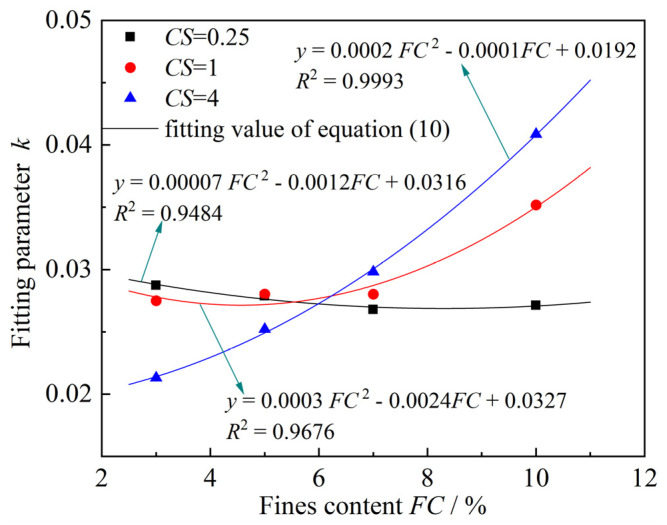
Relationship between *k* and *FC* under different *CS*.

Similarly, the fitting parameter *k* for different clay–silt ratio (*CS*) is plotted against the fines content (*FC*) on the same graph, as shown in Figure 13. It can be observed that a good quadratic function relationship exists between *FC* and *k*. Based on this finding, a function relationship between *FC* and *k* is proposed, as given by Equation (10):

(10) $$k=\psi FC^{2}+\chi FC+\xi$$

In the equation, *Ψ*, *χ* and *ξ* are fitting parameters.

Considering the functional relationships between the fitting parameter *k*, the clay–silt ratio (*CS*), and the fines content (*FC*), along with Equation (7), next we attempt to construct a functional relationship between the void ratio (*e*), the clay–silt ratio (*CS*), the fines content (*FC*), and the consolidation pressure (*p*). That is, we aim to establish a compressibility model for sand–silt–clay mixtures that accounts for the effects of *FC* and *CS*. Since the functional relationship between *CS* and *k* differs for *FC* = 3%, 5% and *FC* = 7%, 10%, these two fines content cases will be considered separately when developing the model.

(1) When *FC* = 3%, 5%, based on Equations (7), (8), and (10), the proposed and established model (11) is as follows:

(11) $$e=e_{0}-\eta_{1}[(CS-CS_{1})+(FC^{2}+FC)]{\left(P/P_{a}\right)}^{\lambda }$$

In the equation, *η*_1_ and *λ* are fitting parameters.

(2) When *FC* = 7%, 10%, based on Equations (7), (9), and (10), the proposed and established model (12) is as follows:

(12) $$e=e_{0}-\eta_{2}[(\ln CS)+(FC^{2}+FC)]{\left(P/P_{a}\right)}^{\lambda }$$

In the equation, *η*_2_ and *λ* are fitting parameters.

Equations (11) and (12) above constitute the compressibility model for sand–silt–clay mixtures that takes into account the effects of *FC* and *CS*.

### 5.2. Validation of the Compression Model for Sand–Silt–Clay Mixtures

**Figure 14:**
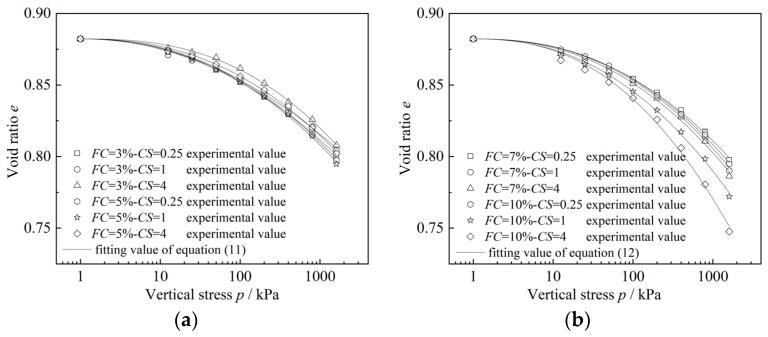
Comparison of the results of the one-dimensional compression consolidation test and model prediction of different specimens in this paper: (**a**) *FC* = 3%, 5%; (**b**) *FC* = 7%, 10%.

**Figure 15:**
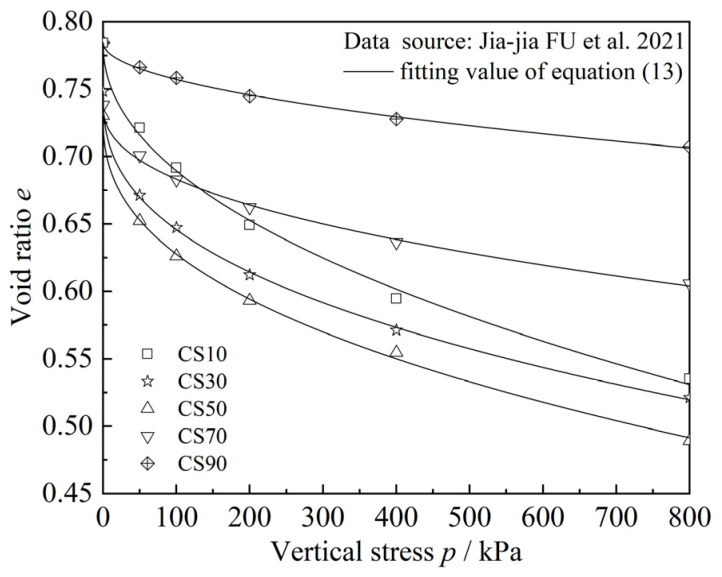
Comparison of the results of the one-dimensional compression consolidation test and model prediction of sand–clay mixtures in reference [11].

**Figure 16:**
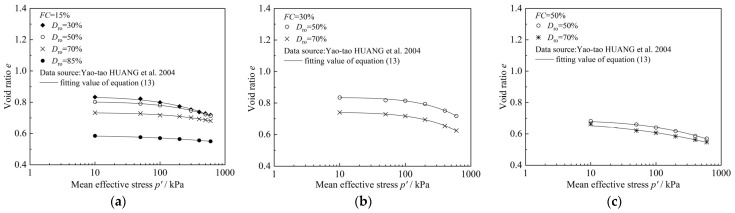
Comparison of the results of the consolidation test and model prediction of sand–silt mixtures in reference [13]: (**a**) *FC* = 15%; (**b**) *FC* =30%; (**c**) *FC* = 50%.

Next, the compression model for sand–silt–clay mixtures is used to validate the experimental results from this study as well as the experimental data from the related literature [11,13]. The specific results are shown in Figure 14, Figure 15 and Figure 16, and the corresponding fitting results are presented in Table 4, Table 5 and Table 6. It should be noted that the specimens used in the experiments from the literature [11,13] are sand–clay mixtures and sand–silt mixtures, with only one type of fines particle present. In such cases, considering the clay–silt ratio (*CS*) is not meaningful. Therefore, when using compression models (11) and (12) proposed in this study for predictions, since the parameter *CS* is not involved, both Equations (11) and (12) can be reduced to Equation (13):

(13) $$e=e_{0}-\eta \left(FC^{2}+FC\right){\left(P/P_{a}\right)}^{\lambda }$$

The experimental data from the literature [11,13] can further be predicted and validated using Equation (13). Firstly, from Figure 14 and Table 4, it can be observed that the predicted results of the newly proposed mixed soil compression model show a good overall agreement with the one-dimensional compression consolidation test results in this study. This indicates that the proposed mixed soil compression model effectively reflects the compression characteristics of different types of sand–silt–clay mixtures. The fitting results in Table 4 show that when using Equations (11) and (12) to predict the one-dimensional compression consolidation test results in this study, the fitting parameters *η*_1_ or *η*_2_ exhibit a relatively large variation range, whereas the fitting parameter *λ* shows a smaller variation range (2.05533~2.60268).

From Figure 15 and Figure 16, as well as Table 5 and Table 6, it can be observed that the mixed soil compression model established in this study is also able to predict the consolidation test results of sand–clay mixtures and sand–silt mixtures from the other literature quite well, demonstrating the validity and applicability of the newly established model. Further comparison reveals that when Equation (13) is used to predict the consolidation test results of sand–clay mixtures from the relevant literature, the fitting parameter *η* varies within a small range (0.01586~0.86301), and the fitting parameter *λ* also shows a relatively small variation range (0.38574~0.50865), as shown in Table 5. However, when Equation (13) is applied to predict the consolidation test results of sand–silt mixtures from the relevant literature, the fitting parameter *η* exhibits a very large variation range (582.08511~37,750,400.00000), while the variation in parameter *λ* remains relatively small (2.29371~5.20503), as shown in Table 6.

Analysis of the fitting parameters from this study and the experimental results from references [11,13] reveals that the fitting parameter *λ* is related to the type of sand used. In this study, the sand particles used are Fujian standard sand, and the fitting parameter *λ* ranges from 2.05533 to 2.60268. In reference [11], the sand used is coastal harbor sand, and the fitting parameter *λ* ranges from 0.38574 to 0.50865. In reference [13], the sand used is fine sand from central and western Taiwan, and the fitting parameter *λ* ranges from 2.29371 to 5.20503. This indicates that different types of sand can lead to variations in the values of *λ*, likely due to differences in particle size, shape, and other geotechnical properties, which affect the consolidation behavior and compressibility of the mixed soils. However, the parameters *η* (*η*_1_, *η*_2_) are related to the fines content. By analyzing the fitting parameters of this study and the experimental results in references [11,13], it can be observed that in this study, as the fines content and the clay–silt ratio increase, *η*₁ and *η*₂ transition from negative to positive. In reference [11], *η* increases as the fines content decreases. In reference [13], under the same relative density *D*r_0_, when the fines content increases from 15% to 30%, *η* increases with the increasing fines content. However, when the fines content further increases from 30% to 50%, *η* decreases as the fines content increases.

## 6. Conclusions

For sand–silt–clay mixtures, one-dimensional compression consolidation tests were conducted under constant initial void ratio conditions to investigate the effects of different fines content (*FC*) and clay–silt ratio (*CS*). Based on the experimental results, *e*–lg*p* (the void ratio (*e*) versus logarithm of the pressure (*p*)) curves showing the variation in void ratio with vertical consolidation stress were obtained. The test results of specimens with the same *FC* but different *CS*, as well as those with the same *CS* but different *FC*, were analyzed. The compressibility of the specimens was explained from the microstructural perspective of particle contact. Subsequently, a mathematical model was preposed that considers the effects of both *FC* and *CS* on the compression characteristics of the mixed soils. The main conclusions are as follows:

(1) When *CS* is constant and *FC* varies, as *FC* increases, the compressibility of the specimens increases continuously, and the compressibility coefficient also increases. At the same time, as *CS* increases, the differences in the *e*–lg*p* curves and compressibility coefficients between different specimens gradually expand.
(2) When *FC* varies, the effect of *CS* on the *e*–lg*p* curve and compressibility coefficient is not consistent. As *CS* increases, for specimens with *FC* = 3%, the compressibility weakens continuously (the compressibility coefficient decreases); for specimens with *FC* = 7% and 10%, the compressibility strengthens continuously (the compressibility coefficient increases); for specimens with *FC* = 5%, there is no clear trend observed in the changes in compressibility and compressibility coefficient.
(3) The distribution state of clay and silt particles in the mixed soil varies under different *FC*. When *FC* = 3%, the silt particles primarily influence the compressive properties of the mixed soil. However, when *FC* = 7% and 10%, the influence of clay particles on the compressive properties becomes more dominant and diversified. In this case, the compressibility of the mixed soil is mainly controlled by the clay particles.
(4) Based on the one-dimensional compression consolidation test results in this study, a compression model for mixed soil considering the effects of *FC* and *CS* was established. This model can effectively reflect the compressive properties of sand–silt–clay mixtures in this study, sand–clay mixtures and sand–silt mixtures from the related literature. The results demonstrate that the model possesses good validity and applicability for sand mixed with two different properties of fines particles (clay and silt) or single property fines particle (clay or silt).

## Figures and Tables

**Table 1:** Basic physical parameters of test materials.

Materials	Effective Grain Diameter (*d*_10_)/mm	Median Grain Diameter (*d*_30_)/mm	Coefficient of Curvature (*C*_c_)	Coefficient of Non-Uniformity (*C*_u_)	Liquid Limit (*w*_L_)/%	Plastic Limit (*w*_p_)/%
Sand particle	0.110	0.132	0.920	1.570	-	-
Silt particle	0.041	0.051	1.020	1.510	-	-
Clay particle	0.003	0.006	0.920	4.330	104	52

**Table 2:** Test program.

Fines Content (*FC*)/%	Fines Particle Ratio			Initial Void Ratio (*e*_0_)	Specimen
	Clay Content (*CC*)/%	Silt Content (*SC*)/%	Clay–Silt Ratio (*CS*)		
3	20	80	0.25	0.8823	*FC* = 3%–*CS* = 0.25
	50	50	1		*FC* = 3%–*CS* = 1
	80	20	4		*FC* = 3%–*CS* = 4
5	20	80	0.25		*FC* = 5%–*CS* = 0.25
	50	50	1		*FC* = 5%–*CS* = 1
	80	20	4		*FC* = 5%–*CS* = 4
7	20	80	0.25		*FC* = 7%–*CS* = 025
	50	50	1		*FC* = 7%–*CS* = 1
	80	20	4		*FC* = 7%–*CS* = 4
10	20	80	0.25		*FC* = 10%–*CS* = 0.25
	50	50	1		*FC* = 10%–*CS* = 1
	80	20	4		*FC* = 10%–*CS* = 4

**Table 3:** Fitting results of different sand–silt–clay mixtures.

Fines Content (*FC*)/%	Clay–Silt Ratio (*CS*)	Fitting Parameter (*k*)	Fitting Parameter (*λ*)
3	0.25	0.02873	0.38921
	1	0.02750	0.38320
	4	0.02131	0.46116
5	0.25	0.02788	0.41152
	1	0.02804	0.41902
	4	0.02521	0.42506
7	0.25	0.02679	0.42209
	1	0.02803	0.43341
	4	0.02982	0.42412
10	0.25	0.02713	0.43161
	1	0.03519	0.41714
	4	0.04087	0.43412

**Table 4:** Fitting results of sand–silt–clay mixtures in this paper.

Specimen	Fitting Parameter (*η*_1_) or (*η*_2_)	Fitting Parameter (*λ*)	R-Squared (*R*^2^)
*FC* = 3%–*CS* = 0.25	−7.61292	2.09450	0.9995
*FC* = 3%–*CS* = 1	−6.60320	2.05533	0.9964
*FC* = 3%–*CS* = 4	−53.99436	2.60268	0.9980
*FC* = 5%–*CS* = 0.25	−5.27461	2.24656	0.9995
*FC* = 5%–*CS* = 1	−5.20440	2.22979	0.9984
*FC* = 5%–*CS* = 4	−7.65558	2.34118	0.9983
*FC* = 7%–*CS* = 025	−183.13191	2.31626	0.9980
*FC* = 7%–*CS* = 1	4523.40726	2.39394	0.9961
*FC* = 7%–*CS* = 4	190.31555	2.32657	0.9941
*FC* = 10%–*CS* = 0.25	−247.63203	2.38420	0.9993
*FC* = 10%–*CS* = 1	2518.96513	2.28276	0.9971
*FC* = 10%–*CS* = 4	342.37367	2.40386	0.9943

**Table 5:** Fitting results of sand–clay mixtures in reference [11].

Specimen	Fitting Parameter (*η*)	Fitting Parameter (*λ*)	R-Squared (*R*^2^)
*CS*10	0.01586	0.50865	0.9977
*CS*30	0.08618	0.38574	0.9994
*CS*50	0.13690	0.40597	0.9989
*CS*70	0.14133	0.42850	0.9974
*CS*90	0.86301	0.47239	0.9965

**Table 6:** Fitting results of sand–silt mixtures in reference [13].

Fines Content (*FC*)/%	Relative Density (*D*_ro_)/%	Fitting Parameter (*η*)	Fitting Parameter (*λ*)	R-Squared (*R*^2^)
15	30	520,026.42535	3.77810	0.9903
	50	2,071,235.63296	4.24299	0.9997
	70	893,172.52201	4.16253	0.9940
	85	5485.43228	2.84228	0.9933
30	50	37,750,400.00000	5.20503	0.9896
	70	9,655,145.79934	4.82729	0.9993
50	50	12,084.05851	3.14643	0.9950
	70	582.08511	2.29371	0.9780

## Data Availability

The original contributions presented in this study are included in this article. Further inquiries can be directed to the corresponding author.
